# Anti-Inflammatory Potential of *Pygeum africanum* Bark Extract: An In Vitro Study of Cytokine Release by Lipopolysaccharide-Stimulated Human Peripheral Blood Mononuclear Cells

**DOI:** 10.3390/ijms25158298

**Published:** 2024-07-30

**Authors:** Agustín Villar, Fredy Silva-Fuentes, Anna Mulà, Andrea Zangara

**Affiliations:** Euromed, S.A., C/ Rec de Dalt 21-23, Mollet del Vallés, E-08100 Barcelona, Spain; avillar@euromed.es (A.V.); fsilvafuentes@euromed.es (F.S.-F.); amula@euromed.es (A.M.)

**Keywords:** *Pygeum africanum*, inflammation, cytokine release, stimulated human peripheral blood lymphocytes

## Abstract

*Pygeum africanum* bark has been shown to inhibit the production of pro-inflammatory prostaglandins in the prostate and reduces the production of leukotrienes and other 5-lipoxygenase (5-LO) metabolites. It has been suggested that inflammation plays an important role in the pathophysiology of benign prostatic hyperplasia (BPH). Data from clinical trials have shown that *P. africanum* improves the symptoms and objective measures of BPH. This in vitro study aimed to assess the anti-inflammatory potential of a proprietary *Pygeum* bark standardized extract (Prunera^®^) on cytokine release from lipopolysaccharide-stimulated human peripheral blood mononuclear cells (PBMCs). PBMCs were obtained from four donors, and a bead-based assay (ProcartaPlex™ panel) was used for the detection and quantitation of cytokines. *Pygeum africanum* bark standardized extract (PABE) induced a statistically significant decrease (*p* < 0.05) of IL-6 in three donors. Other effects were as follows: IL-2 was lowered in all donors in the absence of a clear dose–response relationship; IL-4, IL-5, IL-9, and IL-13 levels were decreased in most donors; IL-22 levels seemed to be suppressed only for donor 4 at lower and medium concentrations; and IL-27 and TNF-α levels decreased at all PABE concentrations in all donors. The anti-inflammatory effect of PABE, particularly the reduction in IL-6 as a marker of inflammation, supports the potential use of this natural compound in the management of BPH and other conditions in which pro-inflammatory cytokines are involved in their underlying pathophysiological mechanisms.

## 1. Introduction

Benign prostatic hyperplasia (BPH) is a histological diagnosis that refers to nodular overgrowth of the epithelium and fibromuscular tissue within the prostate transition zone and periurethral areas. Multiple pleiotropic mechanisms are involved in the prostatic tissue remodeling process [1], but the pathogenesis of BPH is still not fully understood. An investigation of lymphocyte-derived growth factors on prostatic stromal cells showed that BPH tissues expressed interferon-gamma (IFN-γ) and interleukin (IL) IL-2 and IL-4 mRNA, concluding that chronic inflammation may induce an increased growth pattern of fibromuscular tissue in BPH [2]. Other studies further support the link between chronic inflammation and the progression of BPH [3,4]. BPH myxoid nodules are a constant finding in BPH surgical specimens [5], and BPH nodules frequently occur with an interstitial distribution of chronic inflammatory infiltrates mainly composed of chronically activated T-cell lymphocytes and macrophages [6]. A chronic inflammatory state may lead to tissue damage, activate the release of cytokines, increase the concentration of growth factors, and cause a local vicious cycle. The role and the relationship of pro-inflammatory cytokines in chronic inflammation seem to be a critical component of BPH pathogenesis [7]. Accordingly, reducing inflammation has been postulated as an approach to the management of BPH [8].

*Pygeum africanum* Hook. f. (syn. *Prunus africana* (Hook. f.) Kalman), commonly known as the African cherry, is a member of the Rosaceae family of an evergreen species found across the entire continent of Africa. The bark has been exploited for its medicinal properties. *Pygeum* bark contains fat-soluble sterols and fatty acids as its major active components, including phytosterols like beta-sitosterol. These phytosterols possess anti-inflammatory properties that effectively inhibit the prostate gland’s production of pro-inflammatory prostaglandins [9]. *Pygeum* bark also contains pentacyclic triterpenes, ferulic acid, n-docosanol, and tetracosanol, which contribute to reducing prolactin levels and preventing cholesterol accumulation in the prostate. Extracts of *Pygeum* standardized to 13%–14% phytosterols have been extensively researched in animal studies as well as human clinical trials. [9]. *Pygeum africanum* bark extract (PABE) has potentially relevant biological properties, including inhibition of the chemotactic activity of leukotrienes, antagonization of 5-lipoxygenase metabolite production, inhibition of growth factors (basic fibroblast growth factor [bFGF], insulin growth factor-1 [IGF-1], epidermal growth factor [EGF]) and fibroblast proliferation, improvement of prostatic histology, inhibition of the proliferation of prostatic myofibroblasts and fibroblasts, and restoration of prostatic and bulbourethral epithelium secretory activity [10,11,12,13,14].

PABE is one of the common phytotherapeutic agents used to ameliorate low urinary tract symptoms (LUTS) attributable to BPH [15,16]. The proposed mechanisms of action of *P. africanum* bark in LUTS include anti-androgen by inhibiting androgen and progesterone receptors, inhibition of prostate cell proliferation and apoptosis of stromal cells, anti-inflammatory through inhibition of 5-lipoxygenase, and histamine-mediated protective response to the detrusor muscle in the bladder [15]. A 2020 monograph of the European Scientific Cooperative on Phytotherapy (ESCOP) on *Pygeum africanum* bark summarizes the therapeutic indications of this compound for the symptomatic treatment of micturition disorders (dysuria, pollakiuria, nocturia, and urine retention) in BPH (stages I, II, and III as defined by Alken and Vahlensieck) [17]. In a systematic review of 18 studies involving 1562 men with symptomatic BPH, PABE significantly improved urologic symptoms and flow measures [18]. In a multicenter study carried out in central Europe, PABE treatment for 2 months improved domains of the International Prostate Symptom Score (IPSS) questionnaire, quality of life (QoL), and urinary flow parameters [19]. In a cross-sectional study of men with LUTS associated with BPH treated in real-world practice with PABE for 6 months, significant symptom improvement and QoL, as well as patient satisfaction and compliance with treatment, were observed [20]. In a report on *Prunus africana* of the Committee on Herbal Medicinal Products of the European Medicines Agency, based on data from clinical reviews involving a total of 1310 patients and daily doses of 75–200 mg of lipophilic extracts and treatment periods from 15 to 120 days, it was concluded that *Pygeum* bark improved the symptoms and objective measures of BPH and was well tolerated [21].

Cytokines are involved in both the inflammatory process and the epithelial/stromal prostatic cell interactions [22], and given the well-documented effects of PABE on inflammation and cell proliferation, it was considered of interest to assess the effects of its bark extract on cytokine production using an established in vitro model of lipopolysaccharide (LPS)-stimulated human peripheral blood mononuclear cells (PBMCs). As far as we are aware, there are no previously reported in vitro studies using this model to assess the effect of *Pygeum* extracts on a set of pro-inflammatory cytokines. The results of this study will contribute to defining the mechanisms by which PABE improves symptoms of BPH, as shown in clinical studies.

## 2. Results

### 2.1. Viability Assays

**Figure 1:**
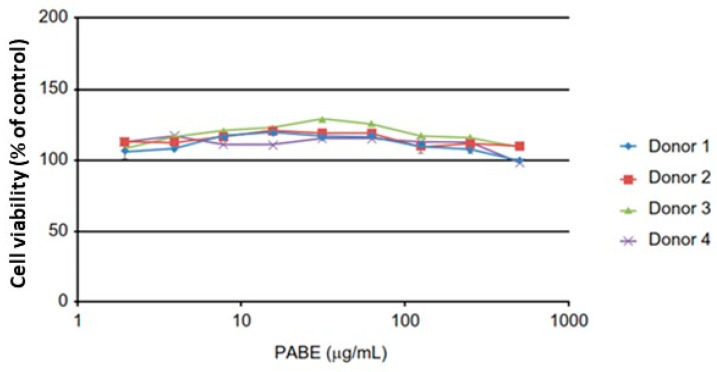
Viability of PBMCs (% concentration) after 24 h exposure to PABE (µg/mL) in each of the four donors. Control cells refer to non-exposed cells.

The viability of the PBMCs was determined by CellTiter-Glo^®^ (CTG, Promega Corporation, Madison, WI, USA) assay after the centrifugation step and withdrawal of the supernatant for cytokine analysis. As shown in Figure 1, PABE had no negative impact on the viability of PBMCs, and all data of the cytokine release assay were valid.

### 2.2. Cytokine Release

The mean (standard deviation, SD) values of changes in the release of cytokines observed in the four donors after exposure to PABE are shown in Table 1.

**Figure 2:**
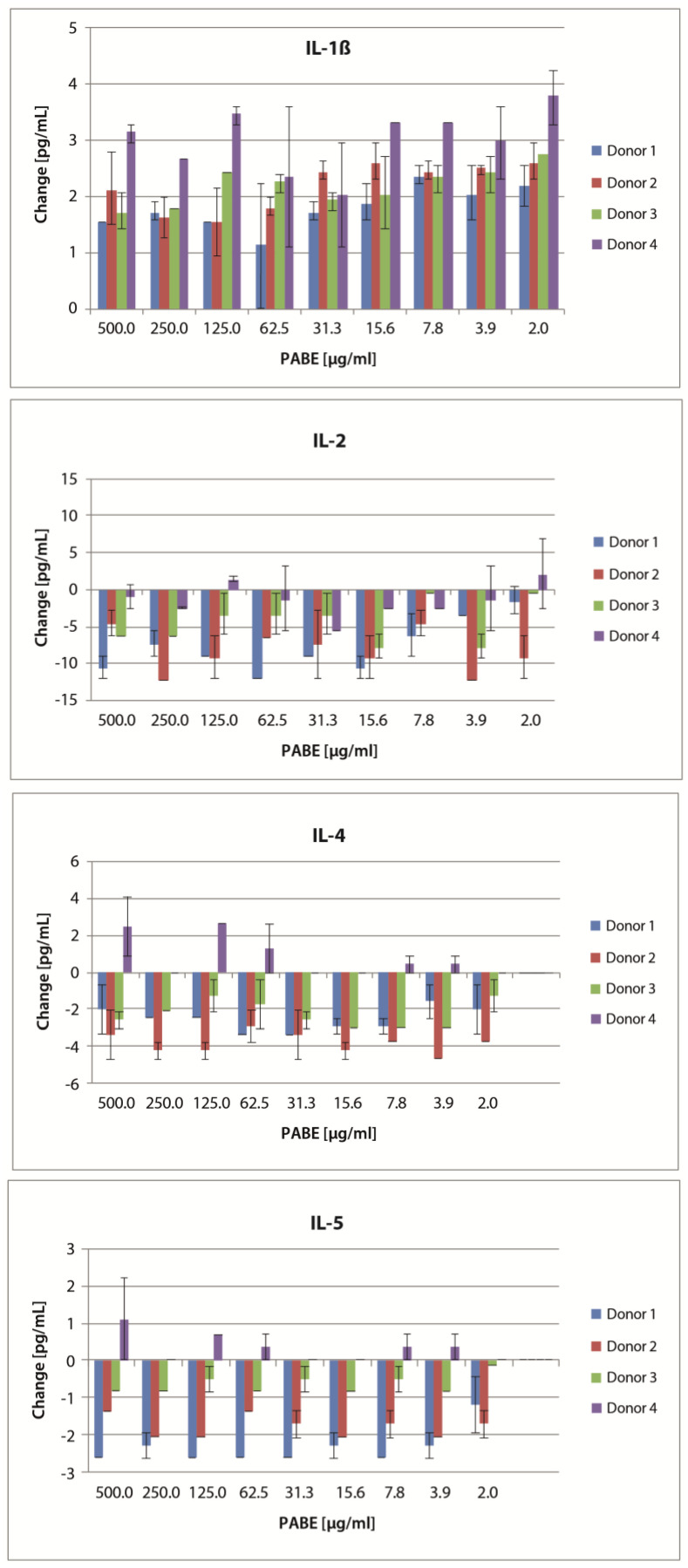

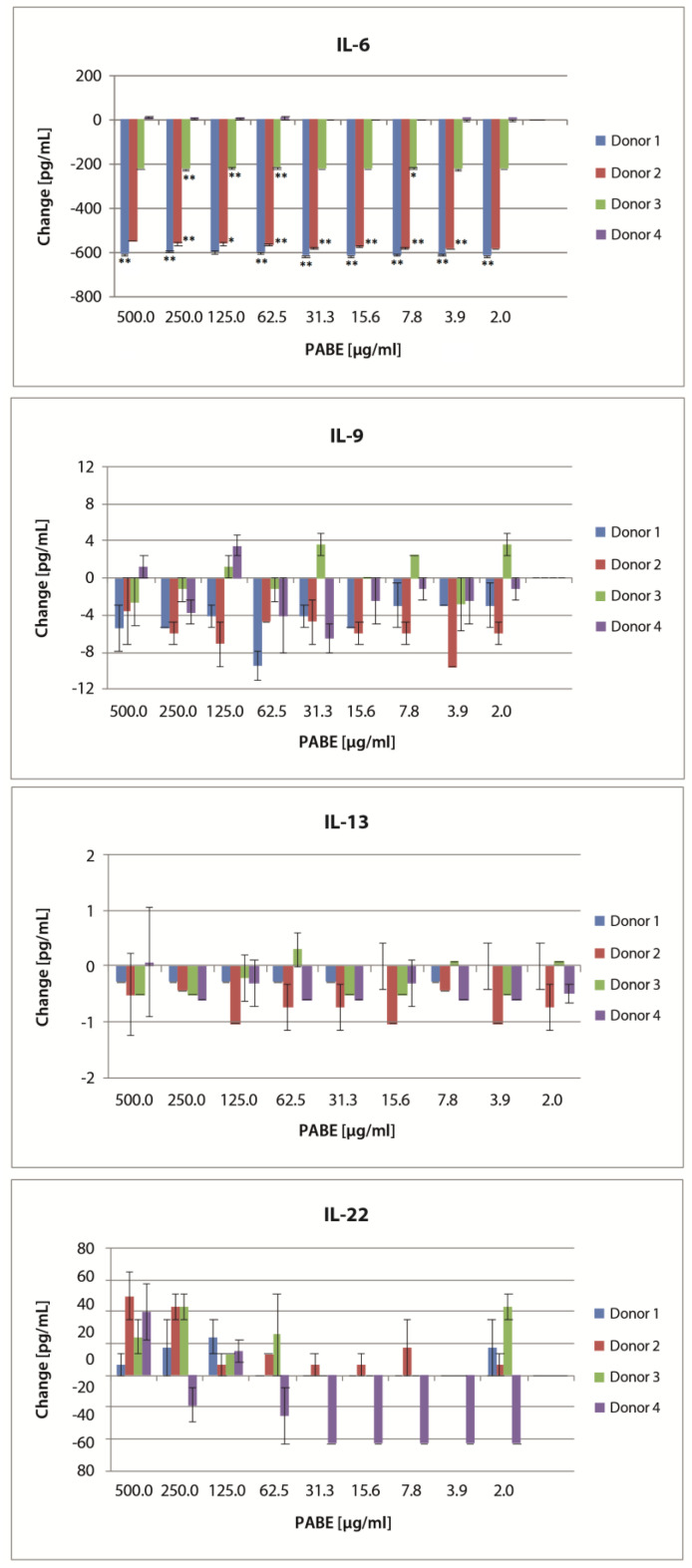

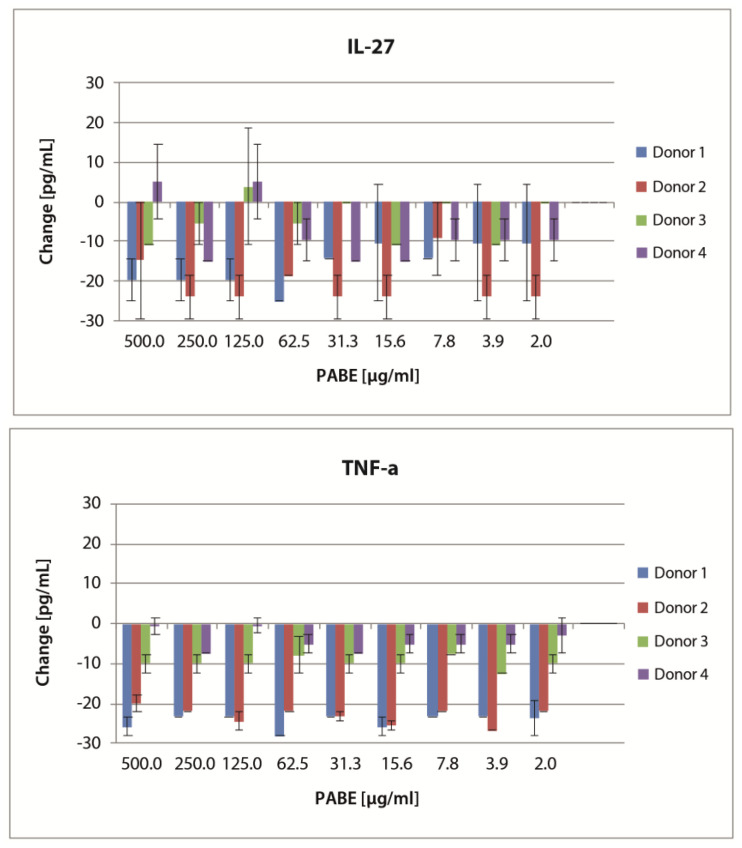
Changes in cytokines IL-1β, IL-2, IL-4, IL-5, IL-6, IL-9, IL-13, IL-22, IL-27, and TNF-α levels (pg/mL) after 24 h exposure to PABE (*Pygeum africanum* bark extract) at different concentrations (500.0 to 2.0 µg/mL). The comparisons between pre- and post-exposure of LPS-stimulated human PBMCs to different concentrations of PABE in the four donors were statistically significant for IL-6 only and for all PABE concentrations in donor 1, for PABE concentrations from 250 to 3.9 µg/mL in donor 2, and for PABE concentrations 250.0, 125.0, 62.5, and 7.8 µg/mL in donor 3, with no significant differences in donor 4. All other comparisons in the remaining panel of cytokines were not statistically significant (* *p* < 0.05, ** *p* < 0.01).

Changes in cytokine levels compared to untreated controls are shown in Figure 2. PABE showed the following effects on the investigated cytokines: changes in IL-1β levels were similar in all four donors. IL-2 was lowered upon exposure to PABE in all donors in the absence of a clear dose–response relationship. IL-4 and IL-5 levels were lower in donors 1–3, whereas donor 4 showed only marginal changes. IL-6 levels were strongly reduced in donors 1–3, while donor 4 showed only minor changes. These decreases in IL-6 levels in donors 1–3 were statistically significant, as indicated in the corresponding panel of Figure 2. Also, IL-9 levels were reduced in donors 1, 2, and 4, while donor 3 showed no clear effects. IL-13 levels were suppressed in all four donors. IL-22 levels seemed to be suppressed only for donor 4 at lower and medium concentrations. Finally, IL-27 and TNF-α levels were lowered at all concentrations in all four donors.

The mean values of the changes in the individual cytokines according to the doses of PABE are shown in Table S1 of the Supplementary Materials.

## 3. Discussion

The present in vitro model provides evidence of the full anti-inflammatory potential of PABE with a trend toward suppression of pro-inflammatory cytokines, although a clear dose–response relationship was not found. However, it may be possible that concentrations of PABE might still be too high to make a biological dose–response apparent. Further studies with lower doses of the active compound are needed to clarify this point. 

The results of the study shed light on the mechanisms by which PABE ameliorates the symptoms of BPH and are, at the very least, suggestive of the potential of this bark extract to be effective in the management of other clinical conditions, the etiology of which may involve a relevant role of pro-inflammatory cytokines. On the other hand, all cytokines selected for our study possess characteristics that contribute to inflammation, cell proliferation, and other pathophysiologic conditions that can be factors in the development of BPH (Table 2).

BPH and associated symptoms of urinary tract outlet obstruction are consequences of hyperproliferation, mainly within the stromal component of the prostate, with increased tonicity of the prostatic smooth muscles. Nodular and fibromuscular hyperplasia are the primary cellular events involved in the pathogenesis of BPH. Studies in cultured prostatic stromal cells obtained from histologically confirmed BPH have shown the antiproliferative effects of *Pygeum*, with inhibition of prostatic myofibroblasts and fibroblasts and induction of apoptosis of stromal cells [14,23]. It has been suggested that the putative molecular basis of inhibition of prostate epithelial and stromal cells includes cell cycle arrest, inhibition of cell signaling pathways at the level of protein kinase C, apoptosis, and binding ability to estrogen and androgen receptors [14].

Growth factors, particularly bFGF, which is found at elevated levels in BPH tissues, appear to play a role in the pathogenesis of BPH. In a study that examined the effects of PABE on basal cell proliferation and on the proliferation induced by bFGF, EGF, and IGF-1, it was found that it had a much larger inhibitory effect on the proliferation of 3T3 fibroblasts when proliferation was induced by 0.5 µg/mL of bFGF, with the effect being significant at 1 µg/mL. PABE also inhibits EGF-induced cell proliferation, but to a lesser extent [24], and inhibition of cell growth induced by certain growth factors may explain, at least in part, the therapeutic effect of PABE in BHP.

Although BPH is primarily characterized by prostatic cell proliferation, inflammation appears to play an important role in the initiation, development, and progression of BPH [22]. Human stromal prostate cells obtained from BPH tissue can actively contribute to the inflammatory process by secreting pro-inflammatory cytokines and chemokines to recruit lymphomononuclear cells [25]. Elevated expression of pro-inflammatory cytokines (IL-6, IL-8, and IL-17) may perpetuate the chronic immune response in BPH and induce fibromuscular growth by an autocrine or paracrine loop or via induction of COX-2 expression [26,27]. In addition, a possible inhibitory effect of *Pygeum* on fibroblast growth factor (FGF-beta 1) and an anti-inflammatory role in neutrophil granulocytes have been reported [28]. A lipophilic extract of PABE inhibits the production of 5-lipoxygenase metabolites, such as chemotactic leukotrienes, in human polymorphonuclear cells stimulated by the calcium ionophore A23187 [29]. 

An important result of this study was a statistically significant difference in the decrease in IL-6 levels after exposure to PABE in the three donors. IL-6 plays a pivotal role in prostate inflammation and has been implicated in the progression of prostate cancer. IL-6 overexpression in prostate tissue has been shown to promote local inflammation, cellular proliferation, and oncogenesis through pathways such as JAK2/STAT3 and IGF signaling [30]. In a prostate-specific IL-6 transgenic mouse model, it was shown that IL-6 induced prostate oncogenesis by amplifying local inflammation [31]. These findings suggest that IL-6 is a critical mediator of the inflammatory milieu of the prostate, contributing to both benign and malignant pathologies. Additionally, elevated IL-6 levels have been observed in patients with untreated metastatic or castration-resistant prostate cancer, correlating negatively with tumor survival and response to chemotherapy [32]. IL-6 facilitates the transition from hormone-dependent to castration-resistant prostate cancer by activating androgen receptor signaling [33].

The results of the present study should be interpreted considering the small sample size, which limits its external validity. However, the large panel of cytokines analyzed is an interesting contribution of this study.

## 4. Materials and Methods

### 4.1. Human Peripheral Blood Mononuclear Cells 

Human peripheral blood mononuclear cells (PBMCs) (ePBMC^®^- uncharacterized cryopreserved human PBMC, size < 10 × 10^6^ cells per vial) were purchased from Cellular Technology Limited (CTL, Shaker Heights, OH, USA) under the auspices of an approved Institutional Review Board (IRB), Quorum Review IRB, Seattle, WA, USA. CTL-certified (6 November 2015) description of the product was as follows: human PBMC isolated from leukopacks and frozen in CTL-CryoABC™ serum-free freezing medium. These leukopacks were ethically collected from four healthy donors (Table 3) with no risk of breaching privacy. All samples tested negative for hepatitis B surface antigen (HBsAg), hepatitis B core antibody (HBcAb), hepatitis C virus (HCV), human T lymphotropic virus types 1 and 2 (HTLV I/II), and standard tests for syphilis (STS) by serology, as well as for human immunodeficiency virus type 1 (HIV-1), HCV, and West Nile virus (WNV) by nucleic acid testing. PBMCs from these 4 donors, 1 vial each, were supplied to Pharmacelsus GmbH (Saarbrücken, Germany) (Pharmacelsus project number 2015 ERM 001, full report 26 January 2016).

A 100× master stock concentration of *P. africanum* bark extract (Prunera^®^, Euromed, S.A., Mollet del Vallés, Barcelona, Spain) was prepared in ethanol at 50 mg/mL. The C_max_ in the final assay was 500 µg/mL, corresponding to 1% ethanol. All subsequent dilutions and controls were prepared using culture media supplemented with 1% ethanol. The stock solution had a brownish appearance with no signs of undissolved particles.

**Figure 3:**
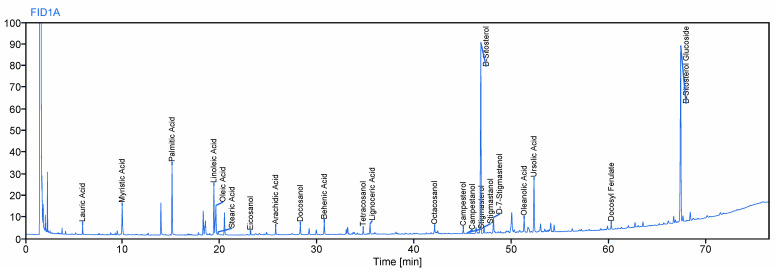
Gas chromatography–flame ionization detector (GC–FID) chromatogram relative to *Pygeum africanum* extract Prunera^®^. Peaks in order of elution: lauric acid, myristic acid, palmitic acid, linoleic acid, oleic acid, steric acid, eicosanol, arachidic acid, docosanol, behenic acid, tetracosanol, lignoceric acid, octacosanol, campesterol, campestanol, stigmasterol, beta-sitosterol, stigmastanol, delta-7-stigmastenol, oleanolic acid, ursolic acid, docosyl ferulate, and beta-sitosterol glucoside.

The test product, Prunera^®^, is a lipid sterol extract derived from the dried bark of the stems and branches of *Prunus africana*, sustainably sourced and certified by the Convention on International Trade in Endangered Species of Wild Fauna and Flora (CITES), ensuring ethical and environmentally responsible practices. It is meticulously standardized to contain no less than 13% beta-sitosterol, determined using a spectrophotometric UV method, which includes saponification and a colorimetric reaction using sulfuric acid, where the total sterols were determined by measuring the absorbance at 510 nm and expressed as units of beta-sitosterol. This extract underwent rigorous analysis by gas chromatography (GC), following the chromatographic method outlined in the United States Pharmacopoeia (USP) *Pygeum* extract monograph [34], to quantify the total sterol content expressed as beta-sitosterol. This CG method entails a quantitative determination of the sterol compounds carried out by high-resolution gas chromatography with flame ionization detection (GC–FID). The procedure comprises the formation of the volatile silyl-derivatives of the sterols and its quantification and identification in front of each commercial standard, using an HT-5 capillary column (25 m × 0.22 mm, 0.1 μm). The injection port was maintained at 300 °C, and the detector temperature was set at 350 °C. Helium was used as the carrier gas. One microliter of the sample was injected at a split ratio of 25:1. A temperature gradient was used, with an initial oven temperature of 150 °C that was increased to 370 °C at 3 °C/min, and the sample was held at this temperature for 15 min. The total run time was 90 min. The internal standard, 5 alpha-cholestane, was used for accurate measurements, and the result was expressed as beta-sitosterol. The analytical results consistently indicated that the total sterol content in the test product exceeded 10%. The method entails the analysis of sterols, including campesterol, stigmasterol, and beta-sitosterol, in *P. africanum* extract (Figure 3). 

### 4.2. Cell Viability Assay

For the detection of viable cells, the CellTiter-Glo^®^ Luminescent Cell Viability Assay (Promega Corporation, Madison, WI, USA) was used following the manufacturer’s specifications. This method determines the number of viable cells in culture based on the quantitation of ATP present as an indicator of metabolically active cells. The procedure involved adding a single reagent (CellTiter-Glo^®^ reagent) directly to cells cultured in a serum-supplemented medium. The homogeneous “add-mix-measure” format results in cell lysis and the generation of a luminescent signal proportional to the amount of ATP present. The cells were cultured in 96-well plates at a volume of 100 µL per well. The CellTiter-Glo^®^ reagent was added to each well in a volume of 100 µL, following the manufacturer’s instructions. Luminescence was detected using a Luminex^®^ instrument platform (Luminex Corporation, Austin, TX, USA), as mentioned in the manuscript. The incubation time after the addition of the reagent was 10 min at room temperature in the dark to stabilize the luminescence signal. For the dose–response relationship, absolute luminescence (background subtracted) was related to the negative (medium) control, and relative viability values were plotted against the test item concentration.

### 4.3. Cytokine Release Assay

Due to the small amount of cytokines released by PBMCs into the supernatant, a bead-based assay (ProcartaPlex™, Santa Clara, CA, USA) using the Luminex^®^ instrument platform (Luminex Corporation, Austin, TX, USA) was used to quantitate 18 different cytokines in parallel within a 50 μL sample using appropriate calibration standards. Human cryopreserved PBMCs were thawed according to the manufacturer’s instructions. After thawing, the cells were allowed to stabilize for 2 h before further processing. Cells were washed, resuspended in RPMI1640 containing 10% fetal bovine serum (FBS), plated in 96-well microtiter plates at 300,000 PBMCs/well, stimulated with lipopolysaccharide (LPS) at 10 µg/mL, and then exposed to different concentrations of PABE for 24 h. The cells were left to stabilize for 24 h after plating in 96-well plates before being treated with LPS. Plates were centrifuged, and cell-free supernatants were collected and forwarded to a cytokine bead-array assay, which was performed according to the manufacturer’s instructions and read using a MagPix multiplex reader.

For the dose–response relationship, absolute concentrations were calculated by MagPix 4.2 software using two separate calibration series, as provided by the manufacturer and summarized in table form.

### 4.4. Statistical Analysis

The mean (standard deviation, SD) values of each individual cytokine released by LPS-stimulated PBMCs before and after exposure to different concentrations of PABE (from 500.0 to 2.0 µg/mL) in each donor were compared with the *t*-test for independent samples. Statistical significance was set at *p* < 0.05. The IBM SPSS statistical software (version 29) was used for the analysis of data.

## 5. Conclusions

The anti-inflammatory effect of *P. africanum* standardized bark extracts, based on inhibition of cytokine release in the in vitro model of LPS-stimulated human PBMCs, supports the potential use of this natural compound in the management of BPH and other conditions in which pro-inflammatory cytokines are involved in their underlying pathophysiological mechanisms.

## Figures and Tables

**Table 1:** Changes in concentrations of cytokines in the four donors after exposure to PABE.

Cytokines pg/mL	Donor 1 Mean (SD)	Donor 2 Mean (SD)	Donor 3 Mean (SD)	Donor 4 Mean (SD)
IL-1β	15.43 (2.81)	19.08 (2.90)	18.95 (1.98)	26.79 (3.86)
IL-2	−68.67 (9.34)	−74.08 (16.70)	−38.14 (11.54)	−12.53 (15.33)
IL-4	−21.15 (3.13)	−30.86 (4.92)	−19.36 (3.13)	7.41 (3.83)
IL-5	−21.20 (1.80)	−16.20 (1.05)	−5.90 (1.05)	2.85 (2.15)
IL-6	−5439.97 (18.48)	−5109.79 (18.09)	−1972.39 (6.58)	31.83 (13.65)
IL-9	−42.65 (11.22)	−53.08 (13.19)	3.24 (11.49)	−16.84 (16.54)
IL-13	−1.76 (1.25)	−6.71 (1.98)	−2.36 (0.73)	−4.15 (1.98)
IL-22	65.52 (52.03)	150.29 (67.43)	148.46 (52.11)	−202.86 (53.73)
IL-27	−144.31 (60.04)	−186.46 (56.07)	−40.19 (25.36)	−73.58 (40.03)
TNF-α	−43.92 (1.86)	−41.39 (1.38)	−17.48 (3.77)	−7.70 (3.65)

**Table 2:** Mechanism of action of the cytokines selected for the study.

Cytokine	Characteristics
IL-1β	Pro-inflammatory cytokine expressed by monocytes, macrophages, and dendritic cells and synthesized in response to inflammatory stimuli (e.g., pathogens).
IL-2	Plays a central role in the activation and proliferation of lymphocytes that have been primed by antigens; pivotal for the expansion of most T-cells, natural killer [NK] cells, and B-cells during various phases of the immune response.
IL-4	Anti-inflammatory cytokine, secreted by activated T-cells and NK cells.
IL-5	Acts as a hematopoietic growth factor, promoting proliferation, activation, and differentiation of certain bone marrow cells.
IL-6	Pleiotropic cytokine, produced mainly by monocytes, plays a central role in host defense mechanisms.
IL-9	Pro-inflammatory cytokine having pleiotropic effects on Th2 lymphocytes, B-cells, and mast cells and implicated in asthma and other allergies.
IL-13	Pleiotropic cytokine expressed by activated T-helper, T-suppressor, and NK cells; suppresses macrophage cytotoxic activity and inflammatory cytokine expression.
IL-22	Regulates production of acute phase proteins of the immunological response; involved in the inflammatory immune response.
TNF-α	Pleiotropic cytokine that plays key roles in innate and adaptive immunity, most often associated with the regulation of pro-inflammatory properties.

**Table 3:** Data from four donors from whom PBMCs were obtained.

Sample Identifier	Gender	Aye, Years	Ethnicity/Race	ABO Group/Rh	Antigen
HHU20130318	Male	38	Asian	A/positive	SFC
HHU20130715	Male	41	Hispanic	0/positive	SFC
HHU20150826	Male	49	Caucasian	Unknown	SFC
HHU20150910	Female	23	African American	Unknown	SFC

PBMCs: peripheral blood mononuclear cells; SFC: spot-forming cells, PBMCs tested in ImmunoSpo^t®^ IFN-γ ELISPOT assay system 400,000 cells/well.

## Data Availability

Data are available from the authors upon request.

## Supplementary Materials

The following supporting information can be downloaded at https://www.mdpi.com/article/10.3390/ijms25158298/s1.
