# Supplementary material for: Anti-Inflammatory Potential of Pygeum africanum Bark Extract: An In Vitro Study of Cytokine Release by Lipopolysaccharide-Stimulated Human Peripheral Blood Mononuclear Cells
Source: Int J Mol Sci. 2024 Jul 30;25(15):8298. doi: 10.3390/ijms25158298 (PMC11311769; doi:10.3390/ijms25158298)
Supplement: Supplementary file 1 [file ijms-25-08298-s001.zip › ijms-3065159-supplementary.pdf]

Table S1. Changes in cytokine levels (IL-1β, IL-2, IL-4, IL-5, IL-6, IL-9, IL-13, IL-22, IL-27, TNF-α) after 24 hours exposure to *Pygeum africanum* bark extract

Cytokines	PABE µg/mL	Donor 1	Donor 2	Donor 3	Donor 4
IL-1β, pg/mL	500	1.46 (0.0)	2.04 (0.68)	1.66 (0.35)	3.13 (0.17)
	250	1.63 (0.17)	1.54 (0.35)	1.66 (0.0)	2.60 (0.0)
	125	1.46 (0.0)	1.44 (0.62)	2.35 (0.0)	3.47 (0.17)
	62.5	1.04 (1.10)	1.72 (0.17)	2.18 (0.17)	2.31 (1.33)
	31.3	1.63 (0.17)	2.39 (0.17)	1.83 (0.17)	1.97 (0.99)
	15.6	1.80 (0.34)	2.56 (0.33)	1.99 (0.69)	3.30 (0.0)
	7.8	2.31 (0.17)	2.39 (0.17)	2.26 (0.25)	3.30 (0.0)
	3.9	1.99 (0.48)	2.45 (0.09)	2.33 (0.33)	2.93 (0.71)
	2.0	2.10 (0.36)	2.56 (0.33)	2.69 (0.02)	3.80 (0.50)
	Control	0	0	0	0
IL-2, pg/mL	500	-10.43 (1.52)	-4.52 (1.79)	-6.10 (0.0)	-0.85 (1.62)
	250	-7.24 (1.67)	-11.97 (0.0)	-6.10 (0.0)	-2.44 (0.04)
	125	-8.91 (0.0)	-9.14 (2.83)	-6.10 (2.83)	1.43 (0.34)
	62.5	-11.96 (0.0)	-6.31 (0.0)	-0.44 (2.83)	-1.17 (4.35)
	31.3	-8.91 (0.0)	-7.35 (4.62)	-6.10 (2.83)	-5.52 (0.0)
	15.6	-10.43 (1.52)	-9.14 (2.83)	-9.15 (1.52)	-2.48 (0.0)
	7.8	-6.08 (2.83)	-4.52 (1.79)	-0.44 (0.0)	-2.48 (0.0)
	3.9	-3.25 (0.0)	-11.97 (0.0)	-9.15 (1.52)	-1.17 (4.35)
	2.0	-1.46 (1.79)	-9.14 (2.83)	-0.44 (0.0)	2.15 (4.62)
	Control	0	0	0	0
IL-4, pg/mL	500	-2.04 (1.34)	-3.36 (1.34)	-2.58 (0.45)	2.49 (1.58)
	250	-2.47 (0.0)	-4.25 (0.45)	-2.13 (0.0)	0.00 (0.0)
	125	-2.47 (0.0)	-4.25 (0.45)	-1.25 (0.88)	2.67 (0.0)
	62.5	-3.37 (0.0)	-2.91 (0.88)	-1.70 (1.34)	1.34 (1.34)
	31.3	-3.37 (0.0)	-3.36 (1.34)	-2.58 (0.45)	0.00 (0.0)
	15.6	-2.92 (0.45)	-4.25 (0.45)	-3.04 (0.0)	0.00 (0.0)
	7.8	-2.92 (0.45)	-3.79 (0.0)	-3.04 (0.0)	0.45 (0.45)
	3.9	-1.58 (0.88)	-4.70 (0.0)	-3.04 (0.0)	0.45 (0.45)
	2.0	-2.04 (1.34)	-3.79 (0.0)	-1.25 (0.88)	0.0 (0.0)
	Control	0	0	0	0
IL-5, pg/mL	500	-2.63 (0.0)	-1.37 (0.0)	-0.85 (0.0)	1.10 (1.10)
	250	-2.28 (0.35)	-2.07 (0.0)	-0.85 (0.0)	0.00 (0.0)
	125	-2.63 (0.0)	-2.07 (0.0)	-0.50 (0.35)	0.70 (0.0)
	62.5	-2.63 (0.0)	-1.37 (0.0)	-0.85 (0.0)	0.35 (0.35)
	31.3	-2.63 (0.0)	-1.72 (0.35)	-0.50 (0.35)	0.0 (0.0)
	15.6	-2.28 (0.35)	-2.07 (0.0)	-0.85 (0.0)	0.0 (0.0)
	7.8	-2.63 (0.0)	-1.72 (0.35)	-0.50 (0.35)	0.35 (0.35)
	3.9	-2.28 (0.35)	-2.07(0.0)	-0.85 (0.0)	0.35 (0.35
	2.0	-1.18 (0.75)	-1.72 (0.35)	-0.15 (0.0)	0.0 (0.0)
	Control	0	0	0	0
IL-6, pg/mL	500	-603.83 (2.51)	-543.72 (0.0)	-218.84 (0.0)	10.13 (3.09)
	250	-594.27 (2.57)	-558.59 (4.68)	-220.04 (1.20)	5.57 (1.47)
	125	-596.74 (6.99)	-558.74 (6.67)	-216.37 (0.76)	7.70 (0.67)
	62.5	-595.26 (0.52)	-563.86 (2.66)	-214.23 (1.37)	6.03 (6.03)
	31.3	-611.32 (2.05)	-574.53 (0.71)	-221.23 (0.0)	0.0 (0.0)
	15.6	-612.18 (1.20)	-572.61 (2.62)	-221.23 (0.0)	0.0 (0.0)
	7.8	-608.51 (0.76)	-576.00 (0.76)	-219.18 (2.05)	0.0 (0.0)
	3.9	-606.38 (1.37)	-580.86 (0.0)	-220.04 (1.20)	1.20 (1.20)
	2.0	-611.48 (0.50)	-580.86 (0.0)	-221.23 (0.0)	1.20 (1.20)
	Control	0	0	0	0
IL-9, pg/mL	500	-5.39 (2.45)	-3.54 (3.54)	-2.55 (2.60)	1.18 (1.18)
	250	-5.34 (0.0)	-5.87 (1.21)	-1.21 (1.25)	-3.66 (1.25)
	125	-4.14 (1.21)	-7.12 (2.45)	1.25 (1.21)	3.51 (1.16)
	62.5	-9.44 (1.59)	-4.67 (0.0)	-1.21 (1.25)	-4.05 (4.05)
	31.3	-4.14 (1.21)	-4.69 (2.38)	3.63 (1.18)	-6.50 (1.59)
	15.6	-5.34 (0.0)	-5.87 (1.21)	0.04 (0.0)	-2.45 (2.45)
	7.8	-2.96 (2.38)	-5.87 (1.21)	2.45 (0.0)	-1.21 (1.21)
	3.9	-2.93 (0.0)	-9.57 (0.0)	-2.80 (2.84)	-2.45 (2.45)
	2.0	-2.96 (2.38)	-5.87 (1.21)	3.63 (1.18)	-1.21 (1.21)
	Control	0	0	0	0

IL-13, pg/mL	500	-0.29 (0.0)	-0.52 (0.73)	-0.52 (0.0)	0.07 (0.97)
	250	-0.29 (0.0)	-0.44 (0.0)	-0.52 (0.0)	-0.62 (0.0)
	125	-0.29 (0.0)	-1.03 (0.0)	-0.22 (0.42)	-0.32 (0.42)
	62.5	-0.29 (0.0)	-0.74 (0.42)	0.29 (0.31)	-0.62 (0.0)
	31.3	-0.29 (0.42)	-0.74 (0.42)	-0.52(0.0)	-0.62 (0.0)
	15.6	0.00 (0.0)	-1.03 (0.0)	-0.52 (0.0)	-0.32 (0.42)
	7.8	-0.29 (0.42)	-0.44 (0.0)	0.07 (0.0)	-0.62 (0.0)
	3.9	0.0 (0.42)	-1.03 (0.0)	-0.52 (0.0)	-0.62 (0.0)
	2.0	0.0 (0.0)	-0.74 (0.42)	0.07 (0.0)	-0.49 (0.17)
	Control	0	0	0	0
IL-22, pg/mL	500	6.75 (6.75)	49.73 (15.05)	24.09 (10.60)	39.84 (17.80)
	250	17.34 (17.34)	42.74 (8.06)	42.74 (8.06)	-18.65 (10.60)
	125	24.09 (10.60)	6.75 (6.75)	13.49 (0.0)	15.05 (6.99)
	62.5	0.0 (0.0)	13.49 (0.0)	25.40 (25.40)	-25.40 (17.34)
	31.3	0.0 (0.0)	6.75 (6.75)	0.0 (0.0)	-42.74 (0.0)
	15.6	0.0 (0.0)	6.75 (6.75)	0.0 (0.0)	-42.74 (0.0)
	7.8	0.0 (0.0)	17.34 (17.34)	0.0 (0.0)	-42.74 (0.0)
	3.9	0.0 (0.0)	0.0 (0.0)	0.0 (0.0)	-42.74 (0.0)
	2.0	17.34 (17.34)	6.75 (6.75)	42.74	-42.74 (0.0)
	Control	0	0	0	0
IL-27, pg/mL	500	-19.73 (5.35)	-14.66 (14.66)	-10.85 (0.0)	5.01 (9.31)
	250	-19.73 (5.35)	-23.98 (5.35)	-5.50 (5.35)	-15.00 (0.0)
	125	-19.73 (5.35)	-23.98 (5.35)	3.81 (14.66)	5.01 (9.31)
	62.5	-25.08 (0.0)	-18.63 (0.0)	-5.50 (5.35)	-9.65 (5.35)
	31.3	-14.38 (0.0)	-23.98 (5.35)	-0.15 (0.0)	-15.00 (0.0)
	15.6	-10.42 (14.66)	-23.98 (5.35)	-10.85 (0.0)	-15.00 (0.0)
	7.8	-14.38 (0.0)	-9.31 (9.31)	-0.15 (0.0)	-9.65 (5.35)
	3.9	-10.42 (14.66)	-23.98 (5.35)	-10.85 (0.0)	-9.65 (5.35)
	2.0	-10.42 (14.66)	-23.98 (5.35)	-0.15 (0.0)	-9.65 (5.35)
	Control	0	0	0	0
TNF-α, pg/mL	500	-5.14 (0.48)	-3.95 (0.43)	-1.99 (0.48)	-0.10 (0.43)
	250	-4.66 (0.0)	-4.38 (0.0)	-1.99 (0.48)	-1.48 (0.0)
	125	-4.66 (0.0)	-4.86 (0.48)	-1.99 (0.48)	-0.07 (0.40)
	62.5	-5.62 (0.0)	-4.38 (0.0)	-1.56 (0.90)	-1.00 (0.48)
	31.3	-4.66 (0.0)	-4.63 (0.25)	-1.99 (0.48)	-1.48 (0.0)
	15.6	-5.14 (0.48)	-5.10 (0.23)	-1.99 (0.48)	-1.00 (0.48)
	7.8	-4.66 (0.0)	-4.38 (0.0)	-1.51 (0.0)	-1.00 (0.48)
	3.9	-4.66 (0.0)	-5.34 (0.0)	-2.47 (0.0)	-1.00 (0.48)
	2.0	-4.71 (0.90)	-4.38 (0.0)	-1.99 (0.48)	-0.57 (0.90)
	Control	0	0	0	0

PABE: *Pygeum africanum* bark extract.
